# The role of macrophages in rosacea: implications for targeted therapies

**DOI:** 10.3389/fimmu.2023.1211953

**Published:** 2023-08-24

**Authors:** Yi Liu, Yin Zhou, Chenyu Chu, Xian Jiang

**Affiliations:** ^1^ Department of Dermatology, West China Hospital, Sichuan University, Chengdu, China; ^2^ Laboratory of Dermatology, Clinical Institute of Inflammation and Immunology, Frontiers Science Center for Disease-related Molecular Network, West China Hospital, Sichuan University, Chengdu, China; ^3^ Medical Cosmetic Center, Chengdu Second People’s Hospital, Chengdu, Sichuan, China

**Keywords:** rosacea, macrophage, inflammation, targeted therapies, skin, immune system

## Abstract

**Introduction:**

Rosacea, a widespread chronic skin condition, may be influenced by macrophages, key immune cells in the skin, although their exact role is not yet fully understood. This review delves into the function of macrophages, their potential contribution to rosacea pathogenesis, current treatments, and promising macrophage-targeted therapies. It concludes by identifying knowledge gaps and potential areas for future rosacea research.

**Method:**

Leveraging systematic and narrative literature review techniques, we conducted a comprehensive search of databases such as PubMed, Embase, and Web of Science. Utilizing keywords like “rosacea” and “macrophages”, we targeted English articles from the last 5 years (2018-2023). We manually checked reference lists of relevant articles for additional studies. We included only articles emphasizing macrophages’ role in rosacea and/or the development of related therapies and published within the specified timeframe.

**Results:**

The systematic search of electronic databases yielded a total of 4,263 articles. After applying the inclusion and exclusion criteria, 156 articles were selected for inclusion in this review. These articles included original research studies, review articles, and clinical trials that focused on the role of macrophages in rosacea and/or the development of macrophage-targeted therapies for the disease. The selected articles provided a comprehensive and up-to-date overview of the current state of research on macrophages in rosacea, including their function in the skin, the potential mechanisms through which they may contribute to rosacea pathogenesis, and the current treatments and therapies available for the disease. Additionally, the articles identified gaps in knowledge regarding the role of macrophages in rosacea and suggested potential areas for future research.

**Conclusion:**

This literature review emphasizes the important role that macrophages, vital immune cells in the skin, may play in the pathogenesis of rosacea, a common chronic inflammatory skin disorder. The selected studies suggest potential mechanisms by which these cells might contribute to rosacea progression, although these mechanisms are not yet fully understood. The studies also spotlight current rosacea treatments and illuminate the promising potential of new macrophage-focused therapies. Despite these insights, significant gaps persist in our understanding of the precise role of macrophages in rosacea. Future research in this area could provide further insights into the pathogenesis of rosacea and contribute to the development of more effective, targeted therapeutic strategies.

## 1 Introduction

Rosacea is a common chronic skin condition with redness, flushing, inflammation, and sometimes visible blood vessels or red, pus-filled bumps. Recent studies reveal variations within the disease spectrum (1–4). These phenotypes include erythematotelangiectatic rosacea (ETR) (3), characterized by persistent facial redness and visible blood vessels; papulopustular rosacea (PPR) (5), characterized by papules, pustules, and occasional nodules; and phymatous rosacea characterized by skin thickening and enlargement, predominantly affecting the nose (rhinophyma) (6). Identifying rosacea phenotypes is crucial for precise diagnosis and personalized management (7). Rosacea, usually appearing in adults over 30, has an unclear pathophysiology (8). Rosacea is influenced by genetics, environment, vascular factors, inflammation, and microbes (9). Rosacea’s prevalence in northern European populations and among those with a family history suggests a genetic predisposition (10). Rosacea can worsen due to environmental triggers like sunlight, heat, spicy foods, alcohol, stress, and certain cosmetics (11). Vascular issues in rosacea lead to facial blood vessel dysfunction, causing persistent redness, flushing, and visible vessels (12). Elevated *Demodex folliculorum* levels in rosacea sufferers suggest its involvement in the disease’s development (13). Rosacea’s pathophysiology involves skin barrier dysfunction and overproduction of proteins such as cathelicidin and kallikrein (14). These proteins cause inflammation, redness, and swelling and can trigger reactions to harmless bacteria (15). Rosacea treatment includes lifestyle changes, topical medication, oral antibiotics, and laser therapy (16). Topical treatments include metronidazole, azelaic acid, ivermectin, and brimonidine. Oral antibiotics like doxycycline are commonly used for their anti-inflammatory effects (17). Macrophages are essential immune cells that protect against pathogens in the skin and throughout the body (18). They ingest and digest pathogens through phagocytosis (19). Macrophages control inflammation, aid in healing and tissue repair, and remove damaged cells. Their role in rosacea is being studied, but their contribution to the disease is likely (20–22). Macrophages release mediators that cause redness, swelling, and pus-filled bumps in rosacea (23). Macrophages promote blood vessel growth, leading to persistent redness and visible vessels in rosacea (24). Macrophages respond to microbial stimuli, including the presence of *D. folliculorum* mites in rosacea patients, exacerbating inflammation (4). Abnormal immune response to environmental triggers may activate macrophages and contribute to rosacea (25). Research on macrophages in rosacea aims to uncover their role and develop new treatments. Understanding their function could reduce inflammation, improve patients’ quality of life, and shed light on related conditions. Further research is required to validate these findings and apply them in clinical practice.

## 2 Method and results

### 2.1 Method

**Figure 1:**
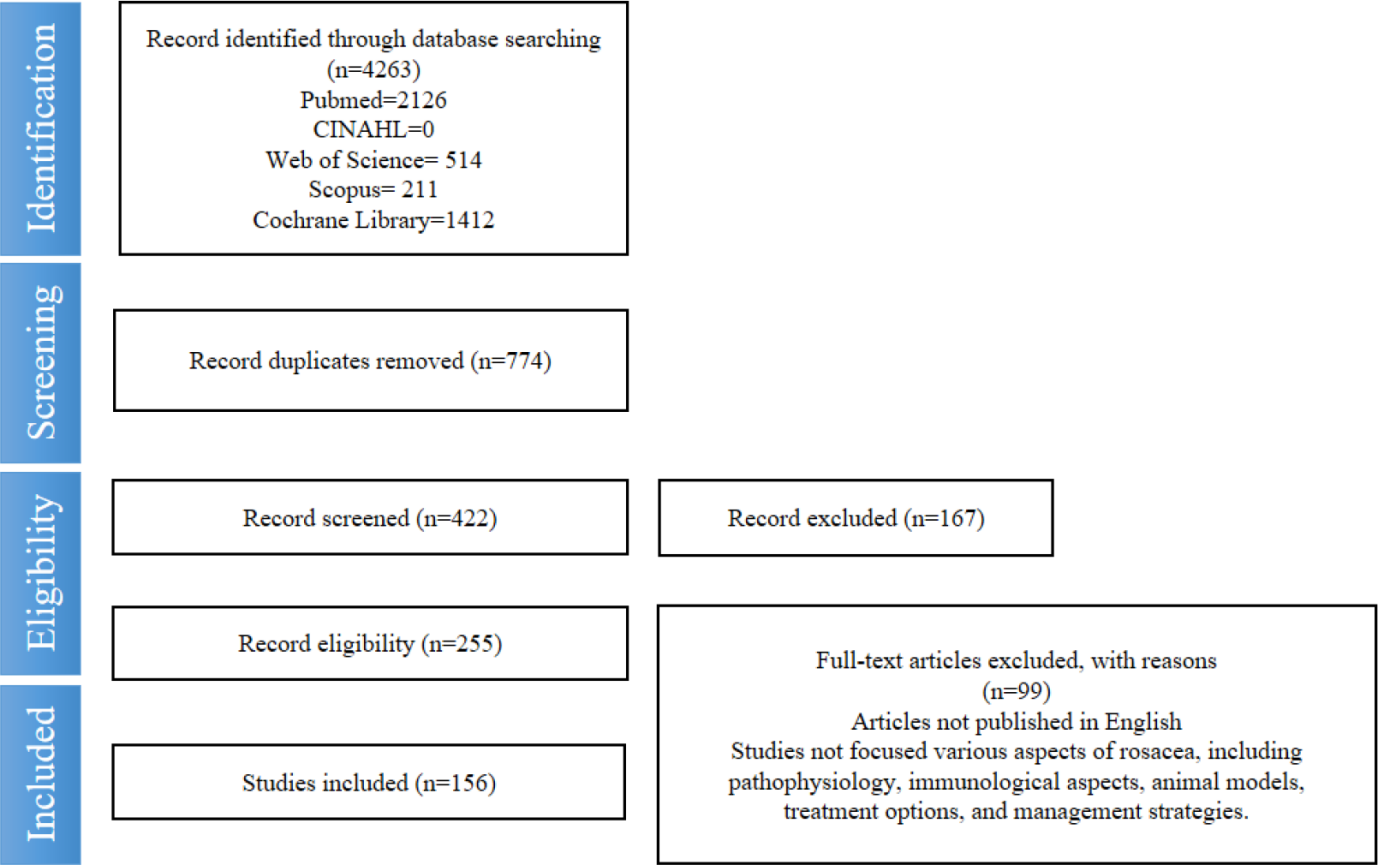
Flowchart according to guideline of PRISMA 2020. PRISMA, Preferred Reporting Items for Systematic Reviews and Meta-Analyses.

The main databases used for the search included PubMed, Embase, and Web of Science. Our search strategy employed specific keywords related to “rosacea” and “macrophages”, with a particular emphasis on English language articles published within the timeframe of 2018 and 2023. Our research methodology extended to manual searches of reference lists from related articles to identify additional studies that could contribute valuable insights to our investigation. To ensure the integrity and relevance of our review, we followed strict inclusion and exclusion criteria, which were determined based on the focus of the article, its relevance to our study, the timeframe of publication, and the language in which the article was written. Our literature review process was the involvement of a multidisciplinary team composed of clinical physicians, dermatology researchers, and immunologists. This diverse team conducted the screening and evaluation process, ensuring a comprehensive and unbiased assessment of the articles based on our predefined criteria. The detailed process of our screening and evaluation, including the specific criteria used, is outlined in the Method section of our study. Flowchart could be referred (**Figure 1**). We believe that this rigorous and comprehensive methodology allowed us to capture a broad and current understanding of the relationship between macrophages and rosacea, contributing valuable insights to the existing body of knowledge.

### 2.2 Results

A systematic search yielded 4,263 articles, with 156 qualifying for inclusion post-criteria application. These articles, encompassing original studies, reviews, and clinical trials, highlighted macrophages’ role in rosacea and potential macrophage-targeted therapies. They provided an updated understanding of macrophages in rosacea, including their functions, contributions to pathogenesis, and current treatments. The articles also identified knowledge gaps and suggested future research areas.

## 3 The role of macrophages in skin and rosacea

Macrophages are immune cells that play a crucial role in maintaining tissue homeostasis and regulating the immune response in the skin (26). Macrophages have several crucial functions, including phagocytosis, debris clearance, antigen presentation, and cytokine secretion to recruit other immune cells to inflammation sites (27). In the skin, macrophages inhabit the dermis and epidermis, interacting with other cells like fibroblasts, keratinocytes, and dendritic cells to sustain skin health (28). In addition to aiding wound healing and tissue repair, macrophages resolve skin inflammation. In rosacea, they are thought to contribute to chronic inflammation and vascular dysfunction (29). Recent advancements underscore the pivotal role of macrophages in rosacea’s pathophysiology (30). Macrophages, acting as scavengers, clear body debris and microbes and are essential for wound healing, tissue repair, and resolving skin inflammation (31). In rosacea, macrophages seemingly contribute to the condition’s hallmark chronic inflammation and vascular dysfunction (32). In rosacea patients, researchers have discovered an overproduction of pro-inflammatory cytokines and angiogenic factors by macrophages (33), which promote inflammation and blood vessel formation. Also, these macrophages exhibit a hindered ability to transition from an inflammatory to a reparative state, extending the inflammatory response and intensifying tissue damage (34). This updated understanding of macrophages’ role in rosacea has unveiled potential therapies, like targeting macrophage function or specific cytokines (35), to alleviate the chronic inflammation and vascular dysfunction associated with the disease. Macrophages have multifaceted roles in rosacea, participating in maintaining skin health and wound healing and contributing to rosacea’s chronic inflammation and vascular dysfunction. A comprehensive overview of these functions and potential therapeutic implications is provided in **Table 1**.

**Table 1:** Role and therapeutic implications of macrophages in rosacea.

Function of macrophages	Description of healthy skin	Changes observed in rosacea	Potential therapeutic implications	References
Presence and interaction	Located in the dermis and epidermis. Interact with fibroblasts, keratinocytes, and dendritic cells for skin health.	No change reported.		(28)
Wound healing and tissue repair	Act as scavengers, clearing debris and microbes. Key for wound healing, tissue repair, and inflammation resolution.			(31)
Inflammatory response and vascular dysfunction	Regulate immune responses including inflammation and vascular functions.	Contribute to chronic inflammation and vascular dysfunction, characterized by overproduction of pro-inflammatory cytokines and angiogenic factors.	Potential targets for alleviating chronic inflammation and vascular dysfunction.	(32, 33)
Transition from inflammatory to reparative state	Capable of switching from an inflammatory state to a reparative state.	Show impaired ability to switch states, leading to prolonged inflammatory response and exacerbated tissue damage.	Aiming to restore this switch could help control rosacea progression.	(34)
Therapeutic target			New understanding suggests potential therapies could target macrophage functions or specific cytokines.	(35)

This table summarizes the key roles of macrophages in maintaining skin health and their contributions to the pathophysiology of rosacea. The final column discusses the potential therapeutic implications based on these functions, highlighting the prospective avenues for rosacea treatment.

### 3.1 Evidence for the involvement of macrophages in rosacea

Studies have shown elevated macrophage levels in the skin of individuals with rosacea, indicating their involvement in the condition (36). Carvedilol effectively treated rosacea by reducing inflammation, improving facial manifestations, and decreasing redness in patients after 4 and 6 months of treatment. It achieved this by inhibiting macrophage TLR2 expression, which may contribute to the vascular dysfunction associated with the disease (37). Studies have shown that macrophages in rosacea-affected skin express elevated levels of pro-inflammatory cytokines like IL-1β and TNF-α, surpassing those found in healthy skin (33). This heightened inflammatory response is thought to contribute to the persistent redness and inflammation seen in rosacea (38). The study examined facial biopsies from rosacea patients, revealing immune system activation and pro-inflammatory cell infiltration across all phenotypes. This prevalent chronic skin disorder presents with diverse signs on the central face, and a standardized system aims to aid diagnosis, research, and health-care communication, underscoring the significance of early identification and treatment to manage symptom progression (2). The updated classification system by the National Rosacea Society improves investigations, diagnosis, and treatment, particularly in specific demographics with a prevalence of 10% or higher, and more frequent diagnoses in women after the age of 30 (39). The efficacy and adverse event rates of various rosacea treatments are summarized in **Supplementary Table 1** (40). Immunohistochemistry and flow cytometry techniques have shed light on the role of macrophages in rosacea by identifying and quantifying these cells in affected skin samples. This is crucial for understanding the immune response in the disease (41). Macrophages may contribute to rosacea pathogenesis through several potential mechanisms (42). One possibility is that they release pro-inflammatory cytokines that contribute to the persistent inflammation seen in the disease (43). These cytokines can trigger immune cell activation and attract more inflammatory cells to the skin, perpetuating an ongoing cycle of inflammation (44). Additionally, macrophages may play a role in the vascular dysfunction seen in rosacea (45). Macrophages are believed to play a role in both angiogenesis (formation of new blood vessels) and vasodilation (widening of existing vessels) in the skin (46), leading to the characteristic redness and flushing of rosacea (47). In rosacea, mast cell activation and the release of matrix metalloproteinases (MMPs) are additional potential mechanisms involved in the disease (48), which can break down the extracellular matrix and contribute to tissue damage (49). Gene expression analysis and functional assays unveil macrophages’ role in rosacea, with observed variations in gene expression profiles between healthy and affected skin samples (5, 50–53). **Table 2** provides an example of such a comparison, highlighting differences in the expression of key genes involved in inflammation and macrophage function.

**Table 2:** Comparison of current treatments for rosacea.

Treatment	Mechanism of action	Dosage	Potential side effects	Effectiveness	Level of evidence
Topical antibiotics (54) (e.g., metronidazole)	Reduces inflammation and bacterial colonization	Apply to affected area twice daily	Dryness, itching, burning	Effective for mild-to-moderate papulopustular rosacea	Randomized controlled trial
Oral antibiotics (55, 56) (e.g., doxycycline)	Reduces inflammation and bacterial colonization	50–100 mg twice daily for several months	Nausea, vomiting, diarrhea, photosensitivity	Effective for moderate-to-severe papulopustular rosacea	Systematic review of clinical trials and meta‐analysis
Topical azelaic acid (57–59)	Reduces inflammation and normalizes skin turnover	Apply to affected area twice daily	Mild burning, stinging, itching	Effective for mild-to-moderate papulopustular rosacea and acne	Systematic review of clinical trials
Topical ivermectin (60–62)	Reduces inflammation and kills *Demodex* mites	Apply to affected area once daily	Burning, itching, dryness	Effective for moderate-to-severe papulopustular rosacea	Randomized controlled studies

## 4 Progress and challenges in macrophage-targeted therapies for rosacea

Current treatments for rosacea typically focus on managing the symptoms of the disease rather than addressing its underlying cause (30). Metronidazole and doxycycline, in topical and oral forms, are commonly used to reduce skin inflammation and bacterial colonization (63). Topical azelaic acid and ivermectin have also been shown to be effective in reducing inflammation and improving the symptoms of rosacea (64). These treatments manage rosacea symptoms but do not address the underlying immune dysregulation and vascular dysfunction associated with the disease (30). Therefore, there is a need for novel, macrophage-targeted therapies that can address the root cause of rosacea (65). A potential macrophage-targeted therapy for rosacea involves using inhibitors that target pro-inflammatory cytokine production (66), such as IL-1β, by macrophages. Anakinra and canakinumab, IL-1β pathway inhibitors with a track record of reducing inflammation in other conditions, hold promise as potential treatments for rosacea (67). Another potential approach is targeting macrophage activation by environmental triggers like UV radiation (68). AhR-modulating drugs can reduce macrophage activation and inflammation in rosacea by targeting the skin’s response to environmental toxins (69). Tapinarof, an innovative topical treatment acting as an AhR agonist, holds promise in treating rosacea (70). This molecular mechanism focuses on the AhR, a ligand-activated transcription factor located in the cytoplasm (71). When tapinarof binds to AhR, it activates the receptor, leading to its translocation into the nucleus of skin cells (70, 72). This triggers the transcription of target genes that regulate inflammatory responses and strengthen the skin barrier function (73). Tapinarof has the potential to alleviate inflammatory responses and vascular dysregulation in rosacea (74, 75). Tapinarof’s potential effectiveness in improving skin barrier integrity may help alleviate rosacea symptoms (70, 76, 77). Referring to current sources is recommended for the latest information, as it may have evolved. *In vitro* assays and preclinical animal models can assess macrophage-targeted therapies’ efficacy and safety for rosacea (78), paving the way for the development of new treatments for this chronic skin disorder (79). Several clinical trials have investigated the efficacy of macrophage-targeted therapies for the treatment of rosacea (65). **Table 3** summarizes trials with anakinra, canakinumab, and an AhR agonist, comparing current rosacea treatments. Topical and oral antibiotics reduce inflammation and bacterial colonization. Topical azelaic acid and ivermectin target skin turnover and *Demodex* mites, respectively. Topical steroids and oral non-steroidal anti-inflammatory drugs (NSAIDs) alleviate redness and inflammation but have potential side effects. Macrophage-targeted therapies show promise in addressing immune dysregulation and vascular dysfunction in rosacea. By targeting the pro-inflammatory cytokines and other molecules produced by macrophages (88), these therapies may be effective in reducing inflammation and improving the symptoms of rosacea (89). Targeted drug delivery systems or immunomodulatory nanoparticles can enhance the efficient and selective delivery of these therapies to the skin, minimizing potential side effects (90). However, there are also several limitations and challenges associated with macrophage-targeted therapies (91). Targeting macrophages specifically while avoiding impact on other skin cell types is challenging due to the complex interactions among immune cells in the skin’s immune response (92). Additionally, the potential for the development of drug resistance and the risk of side effects, such as immunosuppression, must be carefully considered (93). **Table 4** outlines environmental triggers of macrophage activation in rosacea, including UV radiation, temperature changes, stress, alcohol, and spicy foods. These triggers induce inflammation, angiogenesis, nerve sensitivity, and macrophage activation, contributing to the disease’s progression. Furthermore, the cost and availability of these therapies may be a barrier to their widespread use (99). *In vitro* assays, animal models, and clinical trials are valuable for evaluating macrophage-targeted therapies in rosacea and addressing associated challenges (78). Recent progress in understanding the role of macrophages in rosacea has identified macrophage-targeted therapies as a promising treatment approach for the disease (78). However, developing effective macrophage-targeted therapies for rosacea is not without challenges (78). To address these challenges, various techniques have been used to assess the potential advantages and drawbacks of macrophage-targeted therapies for rosacea (78). *In vitro* assays evaluate treatment effects on macrophage function in a controlled environment, while preclinical animal models provide a whole organism setting for testing purposes (100). Clinical trials offer a valuable opportunity to assess the safety and efficacy of macrophage-targeted therapies in human patients (101).

**Table 3:** Comparison of the gene expression profiles of macrophages in healthy *vs.* rosacea skin.

Gene	Expression in healthy skin	Expression in rosacea skin	Reference
IL1B	Low	High	(33, 80–82)
TNF	Low	High	(80, 82–84)
CCL2	Low	High	(82, 85)
CD206	High	Low	(33, 86)
VEGFA	Low	High	(80, 87)

**Table 4:** Summarizing the results of clinical trials of macrophage-targeted therapies in rosacea.

Treatment	Clinical Trial Phase	Number of Participants	Dosage	Duration of Treatment	Outcomes	Reference
Anakinra	Phase II	40	100mg subcutaneously daily	12 weeks	Significant reduction in inflammatory lesion count and erythema	94, 95
Canakinumab	Phase II	36	150mg subcutaneously every 4 weeks	12 weeks	Significant reduction in inflammatory lesion count and erythema	96, 97
AhR Agonist	Preclinical				Significant reduction in macrophage activation and inflammatory cytokine production *in vitro*	70, 98

### 4.1 Transition to a phenotype-based approach in rosacea diagnosis and management

An international group of dermatologists and ophthalmologists has unanimously endorsed a phenotype-based diagnostic and classification system for rosacea, a shift from the traditional approach of consensus-defined primary and secondary features. This new approach primarily identifies two phenotypes, persistent centrofacial erythema and phymatous changes, as independent diagnostic markers, whereas other features such as flushing, telangiectasia, and inflammatory lesions were not considered individually diagnostic. Moreover, the patient-focused transition from subtyping to phenotyping, backed by the ROSacea COnsensus (ROSCO) 2017 recommendations, aims to enhance personalized treatment strategies, taking into account the diverse range of rosacea manifestations and their impact on the patient’s quality of life. The panel also reevaluated treatment modalities based on recent advances in our understanding of rosacea’s pathophysiology, endorsing combination therapies, continued monitoring, and the use of a novel clinical tool, the Rosacea Tracker. These strategic changes aim to promote the utilization of the phenotypes approach in clinical practice and enhance rosacea patient management (47, 102, 103). These techniques reveal the mechanisms and benefits of macrophage-targeted therapies for rosacea. Anakinra and canakinumab showed reductions in inflammatory lesions and erythema in phase II trials, while preclinical studies on AhR agonists demonstrated decreased macrophage activation and cytokine production *in vitro*. Consult **Table 5** for further details on these treatments and clinical trial results.

**Table 5:** Summarizing the known environmental triggers of macrophage activation in rosacea.

Trigger	Mechanism of Activation	Effect on Macrophages	Effect on Skin	Examples	Reference
Ultraviolet radiation	Induces production of reactive oxygen species and pro-inflammatory cytokines	Activates macrophages and increases production of pro-inflammatory cytokines	Promotes inflammation, angiogenesis, and oxidative stress	Sun exposure, tanning beds	36, 45, 104
Temperature changes	Activates sensory neurons that release neuropeptides	Induces vasodilation and increases blood flow, which may activate macrophages	Promotes flushing, inflammation, and nerve sensitivity	Hot showers, exercise	36, 45, 105
Emotional stress	Activates the hypothalamic-pituitary-adrenal axis and sympathetic nervous system	Increases production of stress hormones and pro-inflammatory cytokines	Promotes inflammation and nerve sensitivity	Anxiety, anger, embarrassment	106, 107
Alcohol consumption	Increases blood flow and permeability of blood vessels	Activates macrophages and increases production of pro-inflammatory cytokines	Promotes flushing, inflammation, and nerve sensitivity	Wine, beer, liquor	23, 108
Spicy foods	Activates sensory neurons and increases blood flow	May induce vasodilation and activate macrophages	Promotes flushing, inflammation, and nerve sensitivity	Chili peppers, hot sauce	23, 109, 110
Hot beverages and food	Increasing body temperature and capillary dilation	May increase activity due to elevated body temperature	Can cause flushing, heat sensation	Coffee, Tea, Spicy food	111–113
Certain drugs (vasodilators or nicotinic acid)	Increasing blood flow by dilating blood vessels	Nicotinic acid could impact macrophages directly by modulating inflammation	Vasodilators can cause flushing, nicotinic acid can cause flushing and itching	Nicotinic Acid (Niacin), Nitroglycerin	114–116
Irritation (cosmetic or other topical products)	Topical irritation causes an immune response	Can trigger an inflammatory response	May cause redness, swelling, itching	Certain cosmetics, soaps, lotions	117, 118
Exercise	Increase in body temperature and blood flow	Likely increases activity due to elevated body temperature and increased blood flow	Increased blood flow can cause flushing, sweating	Cardio exercises, Strength training	119, 120

### 4.2 Advancements in understanding macrophage involvement in rosacea

Recent progress has improved our understanding of macrophages’ role in rosacea, a chronic skin condition marked by persistent redness and visible blood vessels (111). Various investigative techniques have been utilized to understand the intricate role of macrophages in the disease (121). One significant method used in this pursuit is immunohistochemistry (122). Immunohistochemistry allows scientists to visualize and assess the distribution and activation state of macrophages in rosacea-affected skin tissue samples using fluorescent or enzyme tags (123). By employing antibodies that target macrophage surface markers like CD68 or CD163, researchers can quantify and identify macrophages at different stages of rosacea progression (33). Flow cytometry, which uses laser light to assess cellular characteristics, is invaluable in determining the phenotype and functional attributes of macrophages (124). Flow cytometry assesses surface markers and cytokine expression to identify macrophage subsets, revealing their roles in triggering inflammation in rosacea (124, 125). Advanced molecular profiling techniques like single-cell RNA sequencing have improved our understanding of the diverse macrophage population (126). Single-cell RNA sequencing is a powerful tool that uncovers gene expression patterns associated with macrophage phenotypes, providing insights into their roles and interactions in rosacea (29, 127). These techniques aid in understanding how macrophages affect rosacea’s development and progression. They pave the way for targeted therapeutic interventions, revolutionizing management by addressing immune responses for more effective treatments in the future.

## 5 Future directions

Despite recent advances, significant knowledge gaps remain regarding the mechanisms by which macrophages contribute to rosacea (128). The environmental triggers that activate macrophages in rosacea, as well as the signaling pathways governing macrophage-mediated inflammation and angiogenesis, remain incompletely understood (129). The heterogeneity of skin macrophages and their interactions with other immune cells and structural cells like fibroblasts are still being investigated (130). Future research should address these knowledge gaps and develop new tools and techniques for studying skin macrophages (131). Advancements in single-cell sequencing, proteomics, and imaging technologies allow for detailed analysis of macrophage phenotypic and functional heterogeneity in the skin (132), as well as their interactions with other cell types. Identifying new macrophage-targeted therapies and improving rosacea treatments rely on these efforts. Recent progress in studying macrophages has paved the way for future research in rosacea (133). One key area of focus will be the development of new macrophage-targeted therapies that can address the underlying immune dysregulation and vascular dysfunction in the disease (134). This may involve identifying new molecular targets for therapy, as well as developing innovative drug delivery systems to improve the efficacy and safety of these therapies (135). Another important area of research will be the use of novel techniques to study macrophage function in the skin (136). Advances in imaging technologies, single-cell sequencing, and other high-throughput techniques may enable a more detailed analysis of macrophage heterogeneity and their interactions with other cells in the skin (137). Various techniques like flow cytometry, single-cell RNA sequencing, and *in vitro* assays, each with unique features and trade-offs, are employed to study macrophage function in rosacea. An overview of these techniques, including their advantages and limitations, is provided in **Table 6**. In addition, the use of preclinical animal models and clinical trials will be critical for evaluating the safety and efficacy of macrophage-targeted therapies and for identifying new molecular targets for therapy (150). Ultimately, the development of new macrophage-targeted therapies and a deeper understanding of macrophage function in rosacea may lead to improved treatments and outcomes for patients with this chronic skin disorder (65).

**Table 6:** An overview of techniques for studying macrophage function in rosacea.

Technique	Principle	Advantages	Limitations
Flow cytometry (82, 138, 139)	Analyzes the expression of surface markers and intracellular molecules in individual cells	Enables analysis of specific cell populations and functional markers	Limited sensitivity for rare populations; requires preparation of single-cell suspensions
Single-cell RNA sequencing (127, 140, 141)	Analyzes gene expression in individual cells	Enables identification of cell subpopulations and gene expression patterns	High cost; requires extensive bioinformatics analysis
Multiplex immunohistochemistry (142–144)	Visualizes multiple markers in tissue sections	Enables spatial analysis of immune cell populations and interactions	Limited to fixed tissue samples; limited number of markers
Intravital imaging (145–147)	Visualizes immune cell behavior in live tissue	Enables analysis of cell behavior in real-time and *in situ*	Limited to superficial tissues; requires specialized equipment
*In vitro* assays (87, 148, 149)	Analyzes macrophage function in culture	Enables precise control of experimental conditions	Limited to artificial conditions; may not reflect *in vivo* function

## 6 Conclusion

In conclusion, recent research has highlighted the potential role of macrophages in the pathogenesis of rosacea. Macrophages are important immune cells that play a critical role in regulating inflammation in the skin, and recent studies have suggested that their dysregulation may contribute to the chronic inflammation and vascular dysfunction seen in rosacea. While current treatments for rosacea focus on managing symptoms, the development of macrophage-targeted therapies represents a promising new approach to treating the underlying cause of the disease. Future research efforts will need to focus on addressing the gaps in our knowledge of macrophage function in rosacea, as well as developing new techniques and therapies to improve patient outcomes. The potential clinical implications of this research are significant, as the development of new macrophage-targeted therapies may lead to more effective treatments for rosacea, a common and chronic skin disorder that can significantly impact patients’ quality of life.

## Author contributions

YL: Writing, concept; CC, YZ: Revise; YL, XJ: Revise, manage the project. All authors contributed to the article and approved the submitted version.

## Supplementary material

Supplementary Table 1 Overview of Rosacea Treatments: Efficacy and Adverse Event Rates.
