# Supplementary material for: The role of macrophages in rosacea: implications for targeted therapies
Source: Front Immunol. 2023 Aug 24;14:1211953. doi: 10.3389/fimmu.2023.1211953 (PMC10484341; doi:10.3389/fimmu.2023.1211953)
Supplement: Supplementary Table 1 — Overview of Rosacea Treatments: Efficacy and Adverse Event Rates. [file Table_1.docx]

| Oral minocycline with topical azelaic acid is just as effective as oral minocycline without azelaic acid | 45 mg minocycline, 15% gel azelaic acid | N/A | Moderate | N/A | N/A |
| --- | --- | --- | --- | --- | --- |
| Topical clindamycin phosphate with tretinoin | 1.2% clindamycin phosphate, 0.025% gel tretinoin | N/A | Moderate | Higher than vehicle/placebo | Moderate |
| Maintenance therapies | | |  |  |  |
| Topical metronidazole 0.75% gel for papules and pustules | 0.75% gel | N/A | N/A | N/A | N/A |
| Topical ivermectin for papules and pustules | 1% cream | N/A | N/A | N/A | N/A |
| Oral doxycycline (40 mg MR) for papules and pustules | 40 mg MR | N/A | N/A | N/A | N/A |
| Recalcitrant rosacea | |  |  |  |  |
| Oral isotretinoin | 10 mg, 20 mg | N/A | No RCTs but recommended by experts | N/A | N/A |
| Oral clindamycin | 150 mg, 300 mg | N/A | No RCTs but recommended by experts | N/A | N/A |

Supplementary Table 1: Overview of Rosacea Treatments: Efficacy and Adverse Event Rates
